# Supplementary material for: Membrane estrogen receptor alpha (ERα) participates in flow-mediated dilation in a ligand-independent manner
Source: eLife. 2021 Nov 29;10:e68695. doi: 10.7554/eLife.68695 (PMC8676342; doi:10.7554/eLife.68695)
Supplement: Figure 5—source data 2. [file elife-68695-fig5-data2.pdf]

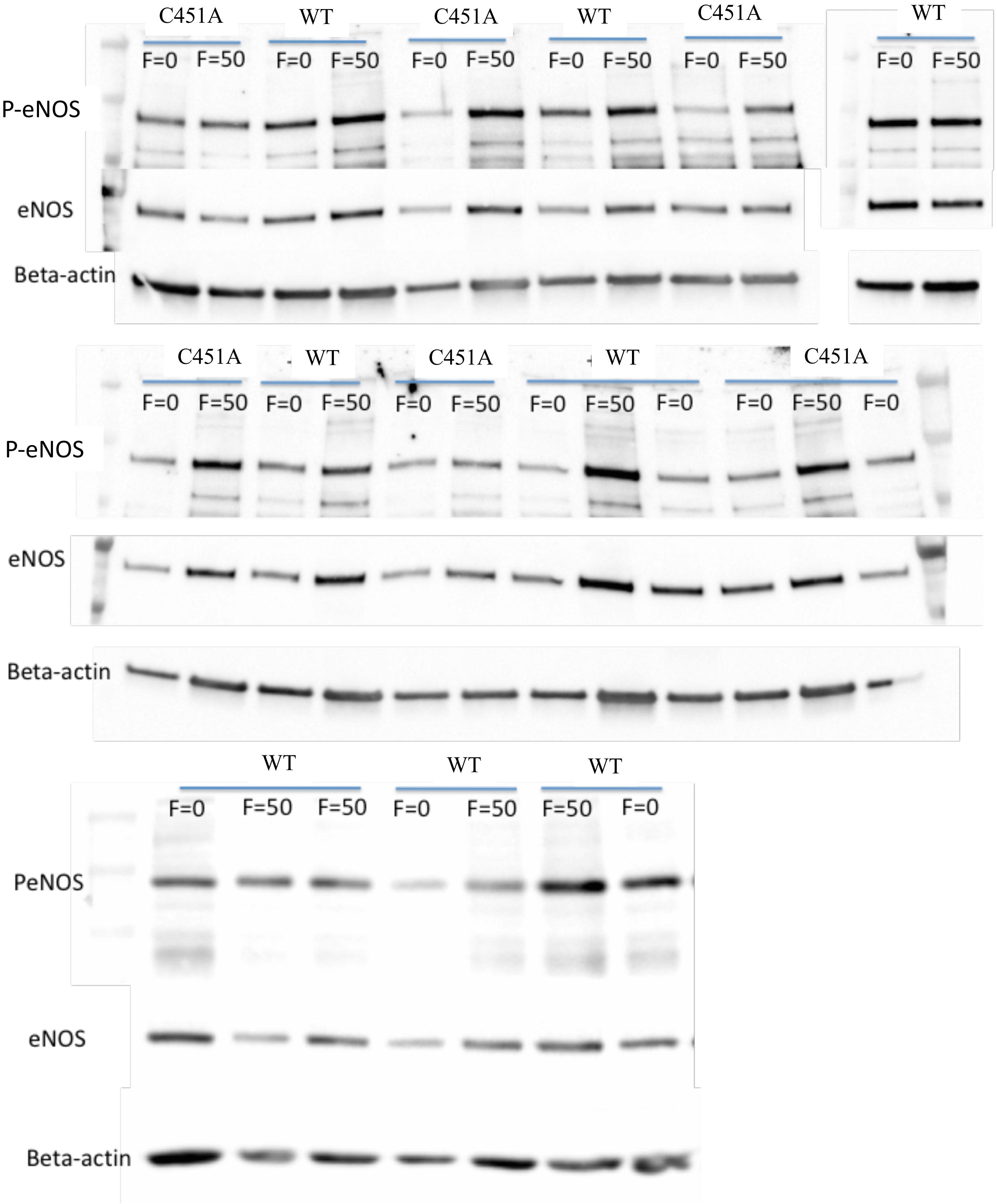

Figure 5: source data 2: Full blots for eNOS, P-eNOS, and beta-actin measured in mesenteric arteries that had been submitted to flow (50 µL/min, F=50) or not (F=0). The arteries were isolated from C451A-ERα (C451A) male mice and their littermate controls (WT).

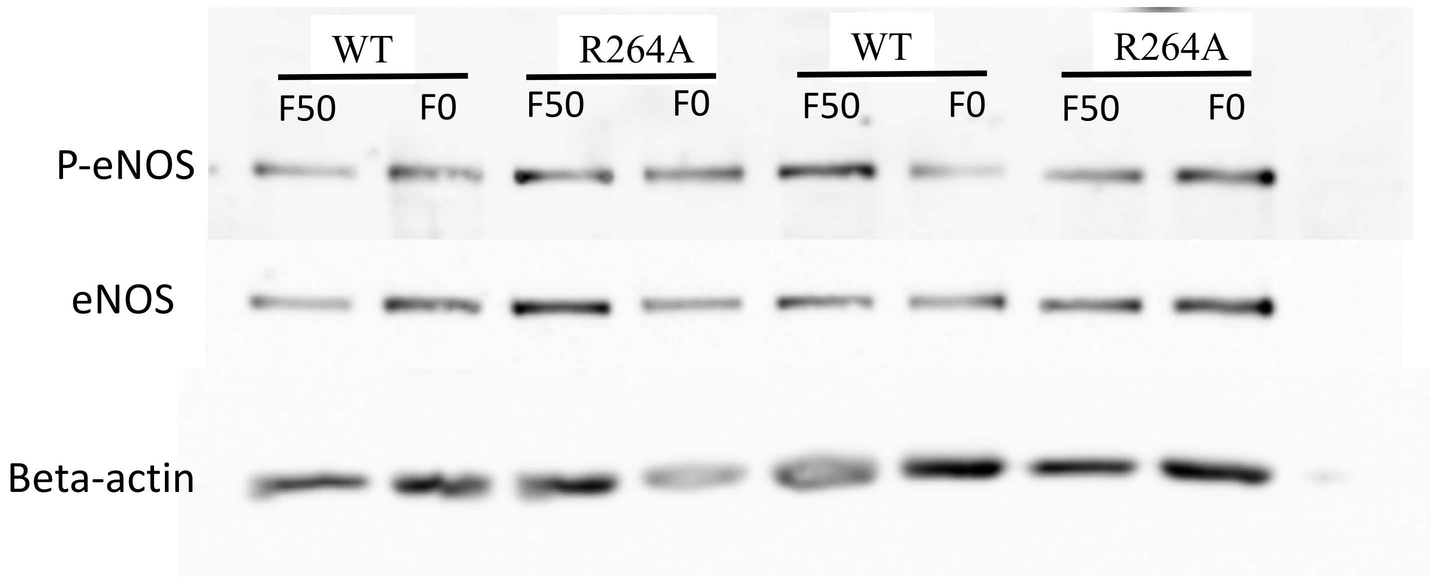

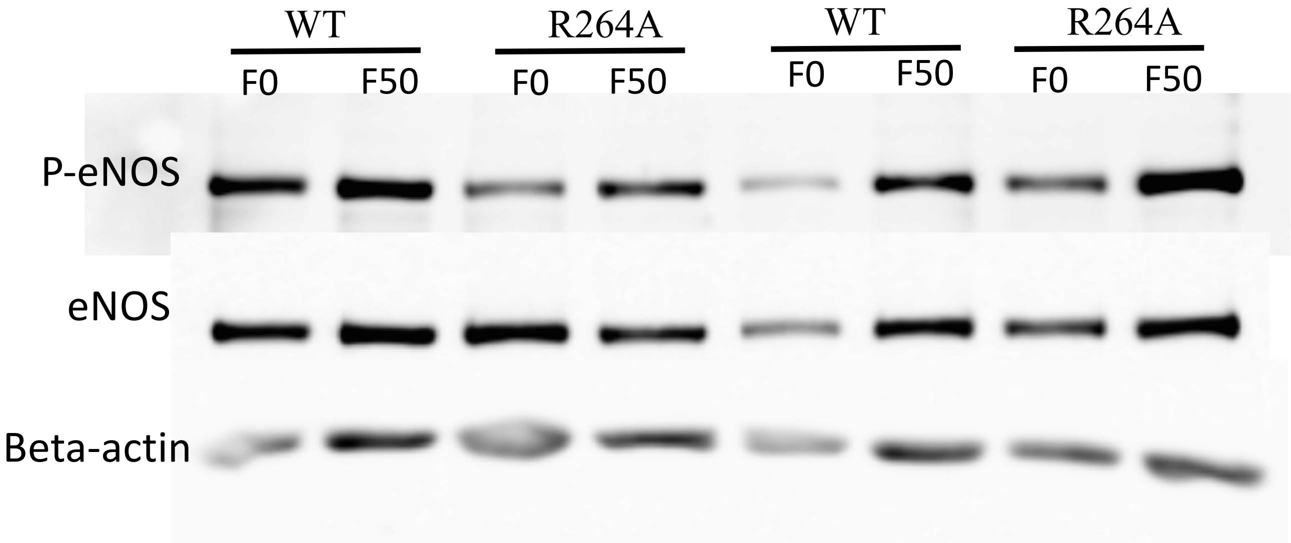

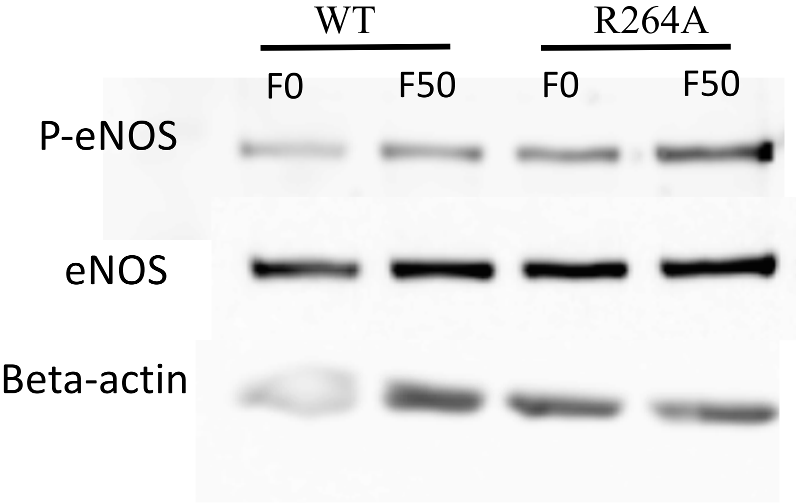

Figure 5: source data 2: Full blots for eNOS, P-eNOS, and beta-actin measured in mesenteric arteries that had been submitted to flow (50 µL/min, F=50) or not (F=0). The arteries were isolated from R264A-ERα (R264A) male mice and their littermate controls (WT).

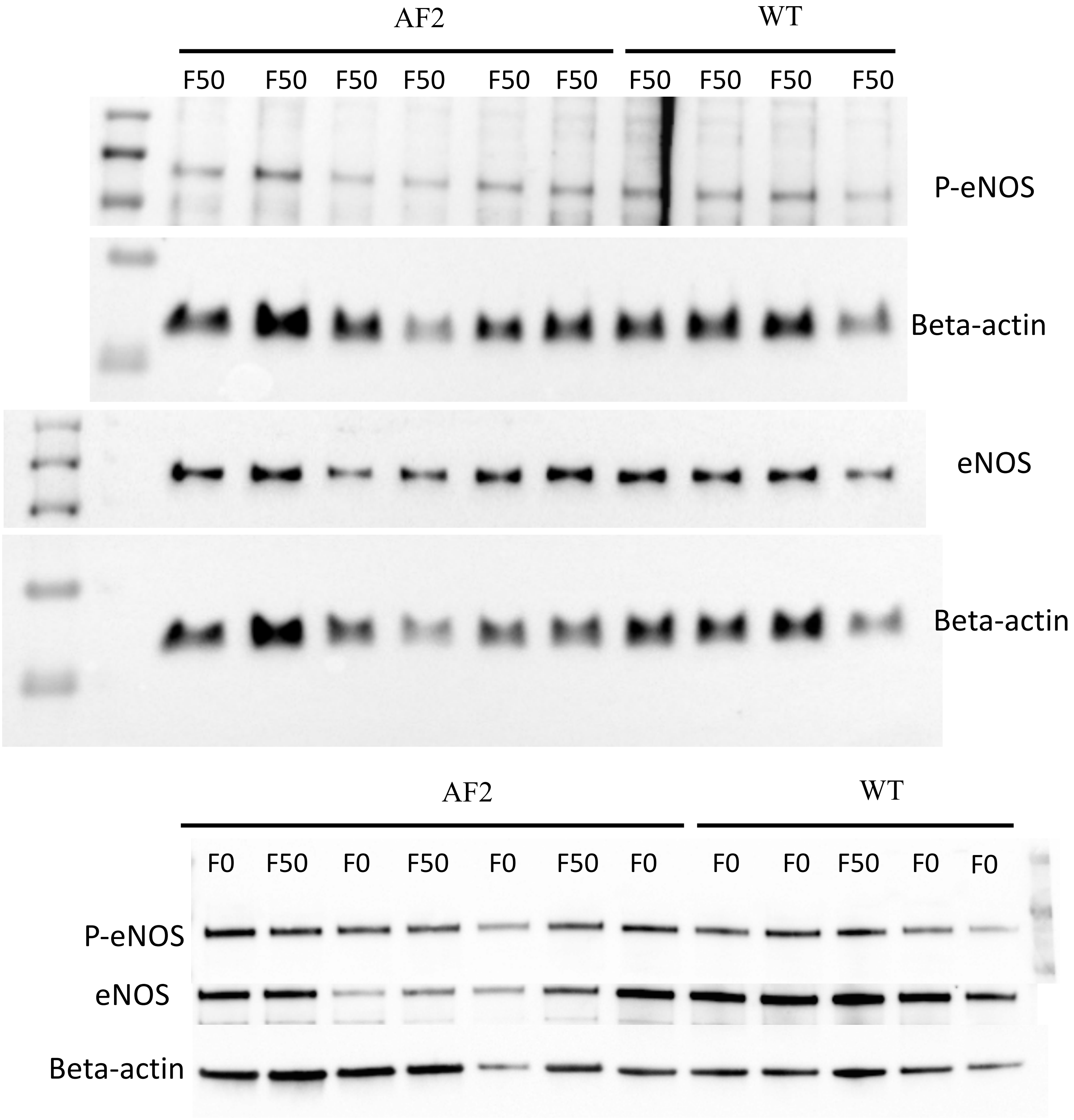

Figure 5: source data 2 : Full blots for eNOS, P-eNOS, and beta-actin measured in mesenteric arteries that had been submitted to flow (50 µL/min, F=50) or not (F=0). The arteries were isolated from AF2-ERα (AF2) male mice and their littermate controls (WT).

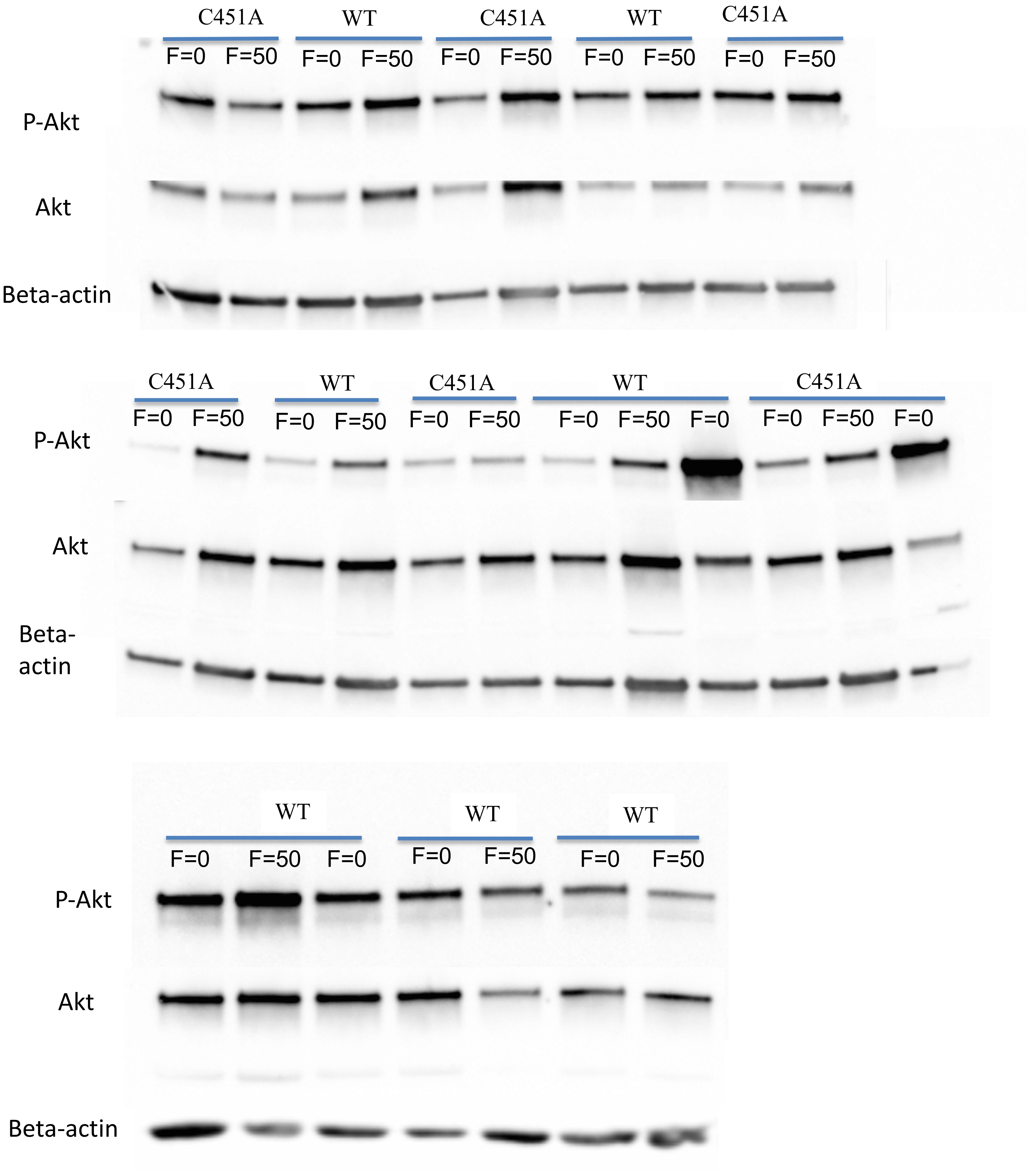

Figure 5: source data 2 : Full blots for Akt, P-Akt, and beta-actin measured in mesenteric arteries that had been submitted to flow (50 µL/min, F=50) or not (F=0). The arteries were isolated from C451A-ERα (C451A) male mice and their littermate controls (WT). Beta-actin similar to eNOS and p-eNOS.

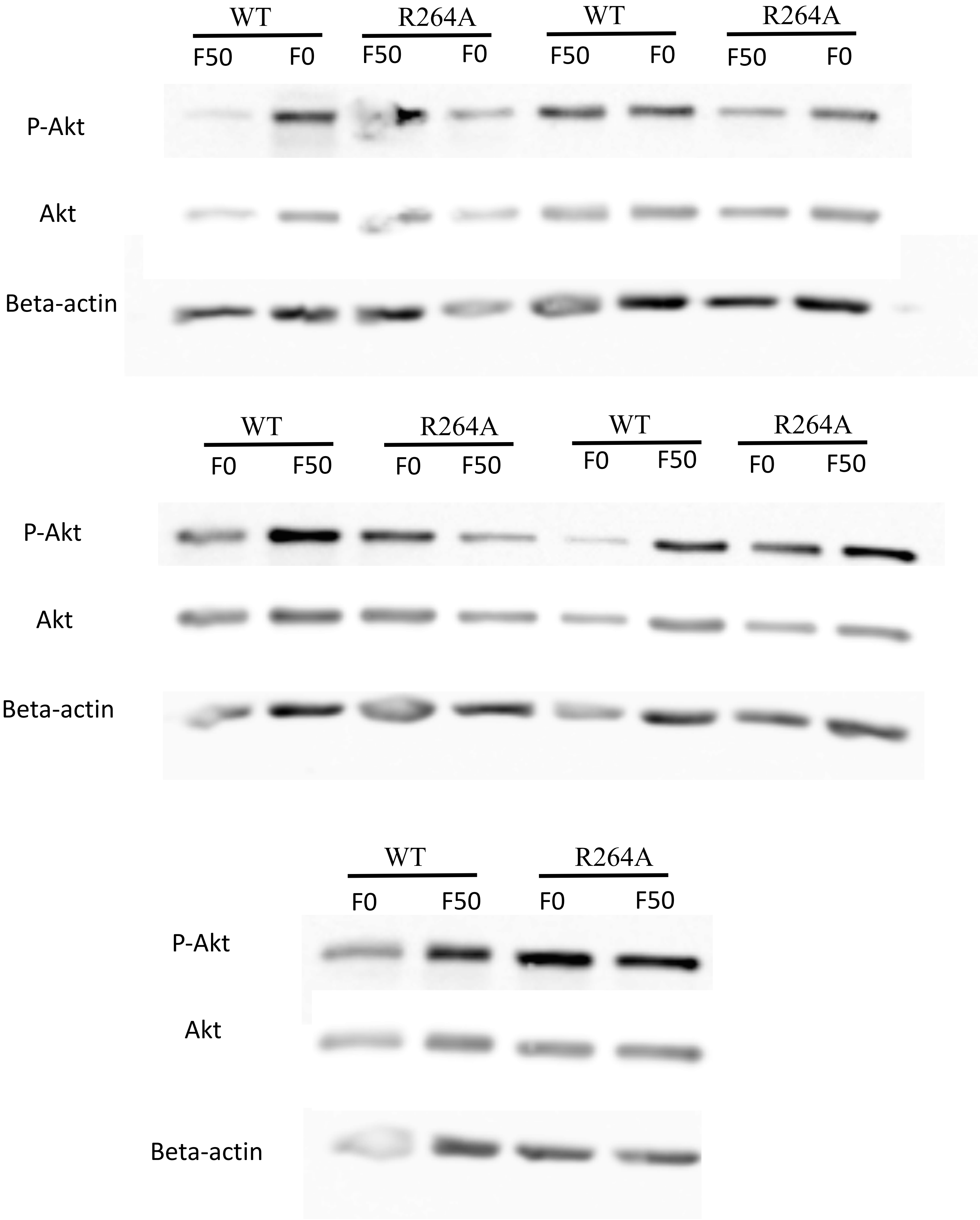

Figure 5: source data 2 : Full blots for Akt, P-Akt, and beta-actin measured in mesenteric arteries that had been submitted to flow (50 µL/min, F=50) or not (F=0). The arteries were isolated from R264A-ERα (R264A) male mice and their littermate controls (WT). Beta-actin similar to eNOS and p-eNOS.

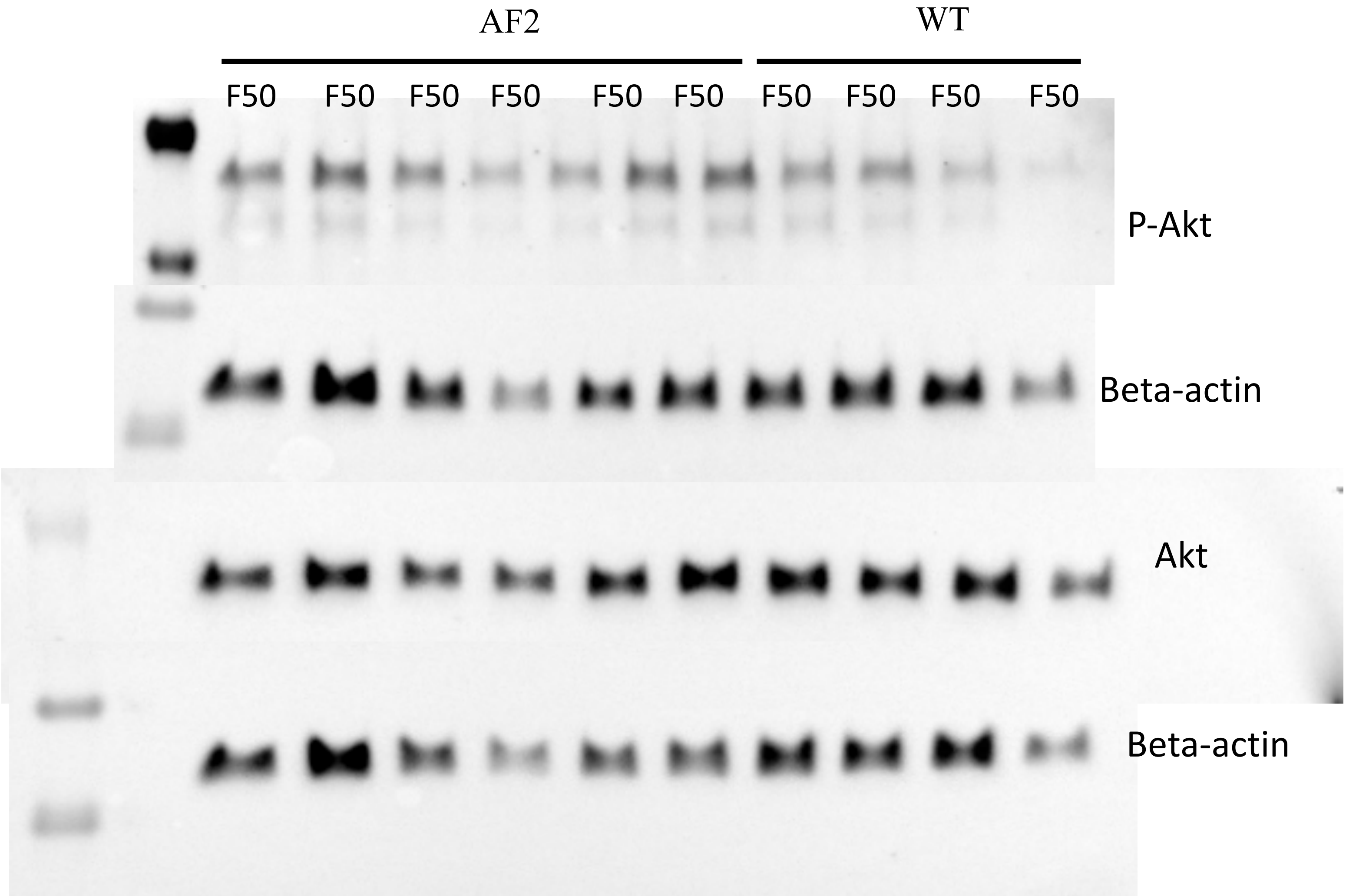

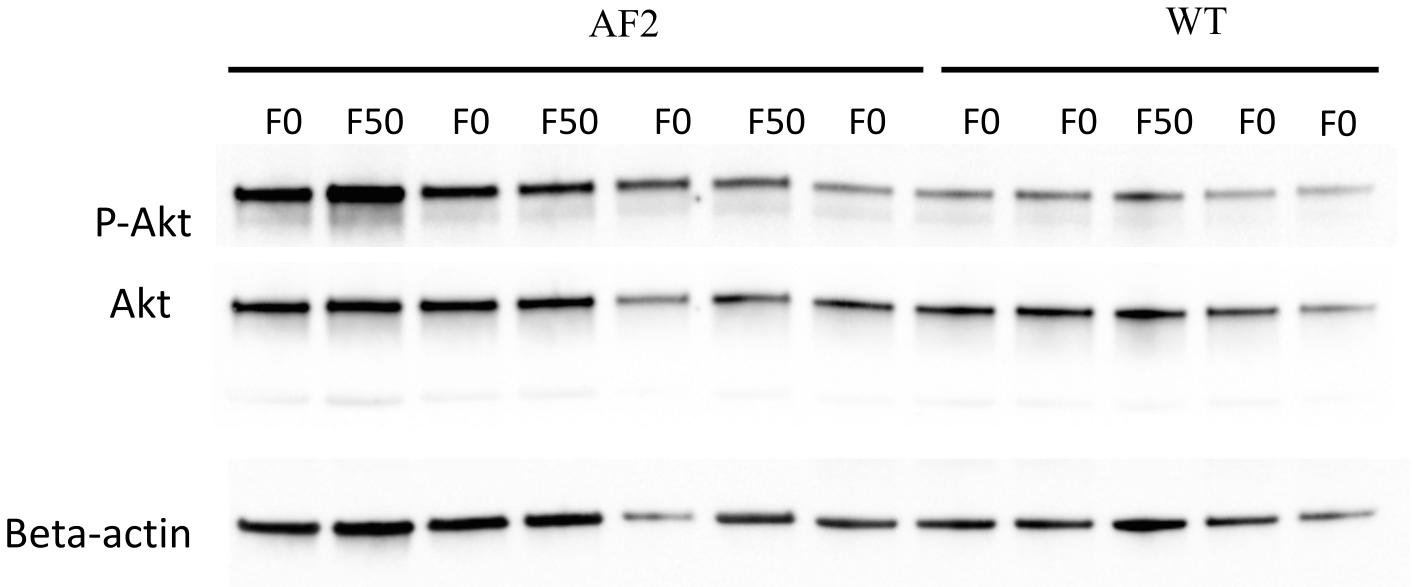

Figure 5: source data 2 : Full blots for Akt, P-Akt, and beta-actin measured in mesenteric arteries that had been submitted to flow (50 µL/min, F=50) or not (F=0). The arteries were isolated from AF2-ERα (AF2) male mice and their littermate controls (WT). Beta-actin similar to eNOS and p-eNOS.
