# Supplementary material for: Membrane estrogen receptor alpha (ERα) participates in flow-mediated dilation in a ligand-independent manner
Source: eLife. 2021 Nov 29;10:e68695. doi: 10.7554/eLife.68695 (PMC8676342; doi:10.7554/eLife.68695)
Supplement: Supplementary file 1. [file elife-68695-supp1.docx]

**Supplementary File 1:**

**Primer sequences used for the RT-qPCR**

Gapdh, Hprt and Gusb were used as housekeeping genes. Analysis was not performed when Ct values exceeded 35. Results were expressed as: 2^(Ct target-Ct housekeeping gene)^.

| **Gene** | **Protein** | **NCBI Reference Sequence** | **Forward sequence (5'-3')** | **Reverse sequence (5'-3')** |
| --- | --- | --- | --- | --- |
| **Agtr1a** | AT1R (a) | NM_177322.3 | actcacagcaaccctccaag | ctcagacactgttcaaaatgcac |
| **Agtr1b** | AT1R (b) | NM_175086.3 | gtgacatgatcccctgacagt | agtgagtgaactgtctagctaaatgc |
| **Mtus1** | AtIP | NM_001005865.2 | tgtgagaagcttcagagcattt | ggcggcgtttaagttgtc |
| **Ace** | ACE | NM_009598.2 and NM_207624.5 | ggaacaagtcgatgttagagaagc | acagaggtacactgcttgatcct |
| **Nos3** | eNOS | NM_008713.4 | ccagtgccctgcttcatc | gcagggcaagttaggatcag |
| **Cav1** | Caveolin1 | NM_007616.2 | aacgacgacgtggtcaaga | Cacagtgaaggtggtgaagc |
| **Esr1** | ER alpha | MN_007956.4 | gctcctaacttgctcctggac | cagcaacatgtcaaagatctcc |
| **Gapdh** | GAPDH | NM_008084.2 | ccggggctggcattgctctc | ggggtgggtggtccagggtt |
| **Gusb** | GUSB | NM_010368.1 | ctctggtggccttacctgat | cagttgttgtcaccttcacctc |
| **Hprt** | HPRT | NM_013556.2 | tgatagatccattcctatgactgtaga | aagacattctttccagttaaagttgag |
| **Icam1** | ICAM | NM_010493.2 | gctaccatcaccgtgtattcg | aggtccttgcctacttgctg |
| **Itga1** | VLa-1, Integrin a1 | NM_001033228.1 | gatggggacgtcaacattct | tgtggttaagacgctaccaaag |
| **Itgb1** | VLa-4, Integrin b1 | NM_010578.1 | atgcaggttgcggtttgt | catccgtggaaaacaccag |
| **Cdh5** | Ve-Cadherin | NM_009868.4 | ggtatcatcaaacccacgaag | ggtctgtggcctcaatgtaga |
| **Tprv4** | TRPV4 | NM_022017.3 | ggcaagagtgaaatctaccagtactat | accgaggaccaacgatcc |
| **Pkd1** | PKD1 | NM_013630.2 | aaacagacctgcttactagcctgt | ctgtctgcagtatcagagtttctgt |
| **Pkd2** | PKD2 | NM_008861.3 | gtgaggacagagctccatttg | gatcccccagtgactgctc |
| **Akt1** | Akt | NM_009652.2 | tcgtgtggcaggatgtgtat | acctggtgtcagtctcagagg |
| **Pdgfa** | Pdgfa | NM_008808.3 | tccagcgactcttggagatag | ccatgggctctcagacttgt |
| **Pdgfb** | Pdgfb | NM_011057.3 | cggcctgtgactagaagtcc | gagcttgaggcgtcttgg |
| **Pparcg1a** | Pgc1a | NM_008904.2 | tgtggaactctctggaactgc | agggttatcttggttggcttta |
| **Tfam** | Transcription factor A, mitochondrial | NM_009360.4 | caaaggatgattcggctcag | aagctgaatatatgcctgcttttc |
| **Mfn1** | Mitofusin 1 | NM_024200.4 | aaaacatactggactcagtaaacgtg | ggtcttccctctcttccattg |
| **Cycs** | Cytochrome C | NM_007808.4 | aacgttcgtggtgttgacc | ttatgcttgcctcccttttc |
| **Shc1a** | p66Shc variant 1 | NM_001113331 | ggacccattctgcctcctct | gccagcttcaggttgctcat |
| **Sod1** | SOD1 | NM_011434.1 | caggacctcattttaatcctcac | tgcccaggtctccaacat |
| **Sod2** | SOD2, mitochondrial | NM_013671.3 | gacccattgcaaggaacaa | gtagtaagcgtgctcccacac |
| **Tie2** | Angiopoietin-1 receptor | NM_013690.3 | ggatgccgccatcaagaggat | ctcggtgttcacatgctccca |
| **Cnn1** | Calponin-1 | NM_009922.4 | ctgttgcgcttgtctgtgtca | gctcccgctgatggtcgtatt |
| **Txnip** | Thioredoxin interacting protein | NM_001009935.2 and NM_023719.2 | gctcgaattgacagaaaagga | accacgattcgggaacac |
| **Mmp2** | MMP2 | NM_008610.2 | gtgggacaagaaccagatcac | gcatcatccacggtttcag |
| **Mmp9** | MMP9 | NM_013599.2 | ttctggcacacgcctttc | ccatagtaagtggggatcacg |
| **Timp1** | TIMP1 | NM_011593.2 | catggaaagcctctgtggat | gatgtgcaaatttccgttcc |
| **Ptgs1** | Cyclooxygenase 1 | NM_008969.3 | cctctttccaggagctcaca | tcgatgtcaccgtacagctc |
| **Ptgs2** | Cyclooxygenase 2 | NM_011198.3 | gggagtctggaacattgtgaa | gcacattgtaagtaggtggactgt |
| **Ptgis** | Prostacycline synthase | NM_008968.3 | aggaaaagcacggtgacatatt | cccacaccactgtgtcgtaa |
| **Sell** | L-Selectin | NM_011346.2 | cagtgtggagcatctggaaa | aaaggctcacactggaccac |
| **Thbs1** | TSP1 | NM_011580.3 | ccccaaccttcccaactc | gggttgtaatggaatggacag |
| **Vcam** | VCAM1 | NM_011693.2 | tgattgggagagacaaagca | aacaaccgaatccccaactt |
